# Human Lung-Resident Macrophages Express and Are Targets of Thymic Stromal Lymphopoietin in the Tumor Microenvironment

**DOI:** 10.3390/cells10082012

**Published:** 2021-08-06

**Authors:** Mariantonia Braile, Alfonso Fiorelli, Daniela Sorriento, Rosa Maria Di Crescenzo, Maria Rosaria Galdiero, Gianni Marone, Mario Santini, Gilda Varricchi, Stefania Loffredo

**Affiliations:** 1 Center for Basic and Clinical Immunology Research (CISI), Department of Translational Medical Sciences, University of Naples Federico II, 80131 Naples, Italy; brailemariantonia@gmail.com (M.B.); mariarosaria.galdiero@unina.it (M.R.G.); marone@unina.it (G.M.); 2 WAO Center of Excellence, 80131 Naples, Italy; 3 Department of Translational Medical and Surgical Science, University of Campania Luigi Vanvitelli, 80131 Naples, Italy; alfonso.fiorelli@unicampania.it (A.F.); rosa.dicrescenzo@unina.it (R.M.D.C.); mario.santini@unicampania.it (M.S.); 4 Department of Advanced Biomedical Sciences, University of Naples Federico II, 80131 Naples, Italy; daniela.sorriento@unina.it; 5 Institute of Experimental Endocrinology and Oncology (IEOS), National Research Council, 80131 Naples, Italy

**Keywords:** angiogenesis, lymphangiogenesis, lung cancer, macrophages, monocytes, monocyte-derived macrophages, thymic stromal lymphopoietin, TSLP isoforms, tumor microenvironment

## Abstract

Thymic stromal lymphopoietin (TSLP) is a pleiotropic cytokine highly expressed by epithelial cells and several innate and adaptive immune cells. TSLP exerts its biological effects by binding to a heterodimeric complex composed of TSLP receptor (TSLPR) and IL-7Rα. In humans, there are two TSLP isoforms: the short form (sfTSLP), constitutively expressed, and the long form (lfTSLP), which is upregulated in inflammation. TSLP has been implicated in the induction and progression of several experimental and human cancers. Primary human lung macrophages (HLMs), monocyte-derived macrophages (MDMs), and peripheral blood monocytes consitutively expressed sfTSLP mRNA. Incubation of HLMs, MDMs, and monocytes with lipopolysaccharide (LPS) or IL-4, but not with IL-13, induced TSLP release from HLMs. LPS, but not IL-4 or IL-13, induced CXCL8 release from HLMs. LPS, IL-4 alone or in combination with IL-13, induced the expression of lfTSLP, but not of sfTSLP from HLMs. Preincubation of HLMs with IL-4, alone or in combination with IL-13, but not IL-13 alone, synergistically enhanced TSLP release from LPS-activated macrophages. By contrast, IL-4, alone or in combination with IL-13, inhibited LPS-induced CXCL8 release from HLMs. Immunoreactive TSLP was detected in lysates of HLMs, MDMs, and monocytes. Incubation of HLMs with TSLP induced the release of proinflammatory (TNF-α), angiogenic (VEGF-A, angiopoietin 2), and lymphangiogenic (VEGF-C) factors. TSLP, TSLPR, and IL-7Rα were expressed in intratumoral and peritumoral areas of human lung cancer. sfTSLP and lfTSLP mRNAs were differentially expressed in peritumoral and intratumoral lung cancer tissues. The TSLP system, expressed in HLMs, MDMs, and monocytes, could play a role in chronic inflammatory disorders including lung cancer.

## 1. Introduction

Thymic stromal lymphopoietin (TSLP) is a pleiotropic cytokine [1,2] highly expressed by lung [3,4,5,6,7,8] and intestinal epithelial cells [9,10,11,12,13,14]. TSLP can be produced also by airway smooth muscle [15] and several immune cells, such as dendritic cells (DCs) [16], mast cells [3,17,18,19], eosinophils [20], and monocytes [16].

Human TSLP exerts its biological activities by binding to a high-affinity heteromeric complex composed of thymic stromal lymphopoietin receptor (TSLPR) and interleukin 7 receptor-α (IL-7Rα) [21]. TSLP initiates signaling by establishing a ternary complex with its specific receptor, TSLPR, and then with IL-7Rα [1,22,23].

Two isoforms (short and long) for TSLP have been identified in different human tissues [9,13]. The short-form TSLP (sfTSLP) is constitutively expressed in normal tissues, including bronchial and intestinal epithelial cells, keratinocytes, and lung fibroblasts [9,13,24,25,26], whereas the long-form TSLP (lfTSLP) is upregulated in inflammatory conditions [9,13]. Despite increasing evidence of a dichotomy for the two isoforms of TSLP in humans, the pathophysiological roles of sfTSLP and to some extent of lfTSLP are largely unknown [2]. There is also evidence that TSLP can be cleaved by endogenous proteases in inflammatory conditions [8,14,27].

The plethora of immune cell types that can either produce or respond to TSLP emphasizes the importance of this cytokine in multiple biological processes [1,2]. A novel and unexpected function of TSLP has been demonstrated in experimental and human cancers [2,28]. In particular, TSLP has been linked to the progression of several experimental [29,30,31,32,33] and human tumors [33,34,35,36,37,38,39,40,41,42,43,44,45,46]. By contrast, few studies have pointed to an anti-tumor role for TSLP in mouse models [47,48,49,50,51] and in human cancers [48]. It is important to emphasize that the differential expression and the functions of the two TSLP isoforms in human and experimental cancers are presently unknown.

Macrophages are important immune cells resident of all tissues [52], where they play pivotal roles in tissue homeostasis [53,54,55]. Tissue macrophages and peripheral blood monocytes represent two branches of the mononuclear phagocyte system, and they have complementary roles during immunological challenges [52]. Macrophages and monocytes are sentinels in immunity, combating infections [56], modulating angiogenesis and lymphangiogenesis [57,58,59], and surveilling agonist tumors [54,60,61,62]. Macrophages arise from different cell lineages emerging during embryonic development [63,64,65,66]. In the lung, tissue-resident macrophages homing during embryogenesis self-renew throughout life [67,68]. During inflammation, bone marrow-derived monocytes can invade the lung and differentiate into macrophages [67]. Macrophages are the predominant immune cells in human lung parenchyma [69,70,71]. Macrophages and monocytes are fundamental regulators of various aspects of tumor immunity [72,73]. In particular, human lung macrophages by producing proinflammatory cytokines (i.e., TNF-α, CXCL8), proangiogenic (i.e., VEGF-A), and lymphangiogenic (i.e., VEGF-C) factors [57,59,74] play a pivotal role in tumor initiation and growth [54].

The expression of TSLP receptor and TSLP isoforms by primary human lung macrophages (HLMs), monocytes and monocyte-derived macrophages (MDMs) has not been characterized. In this study, we evaluated the constitutive and LPS-induced expression of TSLPR, IL-7Rα and TSLP isoforms (sfTSLP and lfTSLP) in HLMs, MDMs, and human peripheral blood monocytes. We also examined the effects of TSLP on the production of angiogenic and lymphangiogenic factors from HLMs and the expression of intratumoral and peritumoral TSLP system (i.e., TSLP receptor and TSLP isoforms) in human lung cancer.

## 2. Materials and Methods

### 2.1. Reagents and Buffers

The following were purchased: bovine serum albumin, L-glutamine, antibiotic–antimycotic solution (10,000 IU/mL penicillin, 10 mg/mL streptomycin, and 25 μg/mL amphotericin B), RPMI 1640, fetal calf serum (FCS) (endotoxin level < 0.1 EU/mL), peroxidase anti-peroxidase, hydrogen peroxide, diaminobenzidine, paraformaldehyde (PFA), Percoll^®^, Triton X-100 (Sigma-Aldrich, St. Louis, MO, USA), detoxified LPS (from *E. coli* serotype 0111:B4), M-CSF, TSLP, IL-13 and IL-4 (Miltenyi Biotec, Bologna, Italy), ELISA kits for TSLP, CXCL8, VEGF-A, VEGF-C, TNF-α (R&D System, Minneapolis, MN, USA), RNeasy plus Minikit (Qiagen, Milan, Italy), high capacity cDNA RT (Life Technologies, Monza, Italy), and iTaqtm Universal SYBR^®^ Green Supermix (Bio-Rad, Hercules, CA, USA). Flow cytometry was performed by the following Abs: anti-CD68 FITC, anti-CD163 FITC, anti-169 PE, anti-CD206 APC, anti-CD24 HV 450 (Miltenyi Biotec, Bologna, Italy), anti-CD5 PE, anti-CD123 APC, HLA-DR HV500, anti-CD22 APC (Becton Dickinson, Italy), anti-CD14 PE-Cy7 (Life-technologies, Monza, Italy), and anti-CD45 APC-Cy7 (BioLegend, Milan, Italy).

### 2.2. Isolation and Purification of Human Lung Macrophages (HLMs)

The study protocol was approved by the Ethics Committee of University of Naples Federico II (Prot. 7/19), and informed consent was obtained from donors. Macrophages were purified from macroscopically normal lung tissue obtained from 39 patients [hepatitis C virus (HCV−), hepatitis B surface Ag (HBsAg−), HIV−] (age 62.4 ± 1.7 years) affected by lung adenocarcinoma undergoing thoracic surgery [59,75]. Freshly resected lung tissue was obtained intraoperatively and was minced finally with scissors and washed extensively with PIPES buffer over Nytex cloth (120-μm pore size (Tetko Elmsford, NY, USA). After Percoll gradient centrifugation, the cells were suspended (10^6^ cells/mL) in RPMI 1640 with 5% FCS, 2 mM L-glutamine, and 1% antibiotic-antimycotic solution and incubated in 24-well plates (Falcon, Becton Dickinson, Milan, Italy). After 12 h, the medium was removed and the plates were gently washed with RPMI. More than 98% of adherent cells were macrophages, as evaluated by flow-cytometric analysis [74].

### 2.3. Flow Cytometry

Human lung macrophages were suspended in PBS at a concentration of 5 × 10^6^ cells/mL. Fifty µL of cell suspension were incubated (20 min at 4 °C) with antibodies. To quench high spontaneous antifluorescence of HLMs, pellets were washed twice with PBS, suspended in 0.2 mL of Crystal violet solution (Certistain, Merck, Damstad, Germany) and incubation of 5 min at 22 °C. Adherent lung cells were examined initially by forward scatter (FSC) area versus side scatter (SSC) area and then by FSC area versus FSC height, with gating on single cells to eliminate dead cells, debris and clumped cells from the analysis. Single cells were then examined by CD45 expression, gating on CD45^+^ cells, which represented total leukocytes. The majority of the adherent lung cells were CD45^+^ leukocytes; within these cells, CD169 (siglec-1), CD206 (mannose receptors), CD68, CD163 and HLA-DR were used to identify macrophages as previously described [70,74]. The vast majority of CD169^+^ cells were human lung macrophages, which were CD206^+^, CD68^+^, CD163^+^, and HLA-DR^+^. The remaining CD45^+^ cells were examined by (1) SSC-A versus CD14 to distinguish CD14^high^ cells, which represent essentially monocytes (0.3%); (2) SSC-A versus CD22 to identify CD22^high^ cells that are B lymphocytes (0.4%); (3) SSC-A versus CD5 to identify CD5^high^ cells which represent T lymphocytes (1.2%). Other minor contaminating cells were granulocytes and monocytes (0.4%) [74]. The samples were acquired by FACS-Canto II and analysed by FACS-DiVa software (Becton Dickinson). Values were expressed as the percentage of positive and negative cells [74].

### 2.4. Isolation of Monocytes and Differentiation of MDMs

The study protocol involving the use of human blood was approved by the Ethics Committee of the University of Naples Federico II, and informed consent was obtained from blood donors (Prot. 301/12). Peripheral blood mononuclear cells were isolated from buffy coats of 32 healthy donors (HCV−, HBsAg−, and HIV−) (age 47.6 ± 2.3 years) obtained from a leukapheresis unit. Leukocytes were separated from erythrocytes by dextran sedimentation [76]. Peripheral blood mononuclear cells (PBMCs) were purified by Histopaque-1077 (Sigma Aldrich, Milan, Italy) density gradient centrifugation (400× *g* for 20 min at 22 °C). Monocytes were further purified with CD14 microbeads according to the manufacturer’s protocol (Miltenyi Biotec, Bologna, Italy). To obtain monocyte-derived macrophages (MDMs), monocytes (1.5 × 10^6^ cells/cm^2^) were differentiated with M-CSF (50 ng/mL) for 7 days in RPMI 1640 supplemented with 10% FCS (Sigma-Aldrich, Milan, Italy) [59].

### 2.5. Cell Incubations

HLMs, monocytes, and MDMs were cultured in 24-well plates in RPMI 1640 supplemented with 5% FBS (Sigma-Aldrich, Milan, Italy), 2 mM l-glutamine, and 1% antibiotic-antimycotic solution.

The cells were treated with IL-13 (10 ng/mL) (Miltenyi Biotec, Bologna, Italy), IL-4 (10 ng/mL) (Miltenyi Biotec, Bologna, Italy), detoxified LPS (100 ng/mL) (from *Escherichia coli* serotype 0111:B4; Sigma-Aldrich, Milan, Italy), or TSLP (5 ng/mL) for 16 h or 6 h at 37 °C. In selected experiments, the cells were preincubated (30 min, 37 °C) with or without actinomycin D (1 μg/mL) and then stimulated (16 h, 37 °C) with LPS or IL-4. At the end of incubation, the supernatants were collected and stored at −80 °C for subsequent ELISA quantification of cytokines. Lysis of the cells in the plates was carried out by using 0.1% Triton X-100 for total protein quantification by a Bradford-based assay (Bio-Rad, Segrate, MI, Italy).

### 2.6. mRNA Extraction and Quantitative PCR (qPCR) Analysis

Total RNA was isolated with RNeasy plus Minikit (Qiagen, Milan, Italy) following manufacturer’s instructions. RNA quality and integrity was estimated with 2100 Agilent Bionalyzer. Total mRNA was reverse-transcribed (high capacity cDNA RT, Life Technologies, Monza, Italy) and quantitative RT-PCR was carried out in Master Cycler realplex (Eppendorf, Milan, Italy) using iTaqtm Universal SYBR^®^ Green Supermix (Bio-Rad, Hercules, CA, USA). GAPDH was used as housekeeping gene to normalize Ct (cycle threshold) values using the 2-ΔCt formula. The following primer pairs were used: GAPDH: forward, 5′-GTCCACTGGCGTCTTCAC-3′ and reverse, 5′-CTTGAGGCTGTTGTCATACTTC-3′; sfTSLP: 5′-CCGCCTATGAGCAGCCAC-3′ and 5′-CCTGAGTAGCATTTATCTGA-3′; lfTSLP: 5′-CACCGTCTCTTGTAGCAATCG-3′ and 5′-TAGCCTGGGCACCAGATAGC-3′; TSLPR: 5′-AGAGCAGCGAGACGACATTC-3′ and 5′-CCGGTACTGAACCTCATAGAGG-3′, IL-7Rα: 5′-TCGCAGCACTCACTGACC-3′ and 5′-CGGGAAGGAGCCAATGAC-3′. Target-specific primers for sfTSLP, lfTSLP, TSLPR, IL-7Rα, and GAPDH were produced and purified by Custom Primers (Life Technologies, Milan, Italy).

### 2.7. ELISA Assays

Cytokine concentrations in supernatants and in cellular lysates were measured using commercially available ELISA kits for TSLP (31.2–2000 pg/mL), CXCL8 (31.2–2000 pg/mL), VEGF-A (31.3–2000 pg/mL), VEGF-C (109–7000 pg/mL), TNF-α (15.6–1000 pg/mL) (R&D System, Minneapolis, MN, USA). Since the number of adherent macrophages and MDMs can vary among the wells and different experiments, the results were normalized for the total protein content in each well, determined in the cell lysates (0.1% Triton X-100) by the Bradford assay. Cytokine release was expressed as pg of specific cytokine/mg of total proteins [77].

### 2.8. Cytospin

Cytospin of HLMs (3 × 10^4^ cells) was done in PBS containing 0.5% albumin by centrifugation (800 rpm, 3 min, 22 °C) onto microscopic slides using a Shandon Cytospin 3 Cytocentrifuge (Shandon, Astmoor, UK). Slides were allowed to dry and stained with Diff-Quich (Biomap, Agrate Brianza, MB, Italy).

### 2.9. Human Lung Tissue and HLM Immunohistochemistry

Immunohistochemistry was performed as previously described (Sorriento, Molecular Cancer 2019). Peritumoral and intratumoral lung tissues were fixed in 10% buffered formalin and embedded in paraffin. Paraffin-embedded sections were processed for immunohistochemistry by peroxidase anti-peroxidase method using as primary antibody rabbit polyclonal anti-TSLP antibody (1:100) (PA5-78610), rabbit polyclonal antibody anti-TSLP Receptor (PA5-203789, or rabbit polyclonal antibody anti-IL-7Rα (1:100) (PA5-97870) (Invitrogen, Thermo Fisher Scientific, Monza, Italy). The secondary antibody was a goat anti-rabbit IgG (GtxRb-003-DHRPX, ImmunoReagents, Milan, Italy). The peroxidase was revealed in presence of 0.03% hydrogen peroxide and of the electron donor (2.5% diaminobenzidine), which becomes visible as a brown precipitate. For negative controls, the primary antibody was omitted. Sections were then viewed with an Eclipse E1000 Fluorescence Microscope (Nikon) and acquired using Sigma Scan Pro software (Jandel). For immunocytochemistry analysis in HLMs, cells were cytospinned on microscope slides and processed as described above.

### 2.10. Statistical Analysis

The data are expressed as mean values ± SD of the indicated number of experiments. Statistical analysis was performed in Prism 6 (GraphPad Software). Statistical analysis was performed by Student’s T-test or one-way analysis of variance followed by Dunnett’s test (when comparison was made against a control) or Bonferroni’s test (when comparison was made between each pair of groups) by means of Analyse-it for Microsoft Excel, version 2.16 (Analyse-it Software, Ltd., Leeds, UK). Values of *p* < 0.05 were considered significant.

## 3. Results

### 3.1. TSLP Isoforms and TSLP Receptor in Human Lung Macrophages

**Figure 1:**
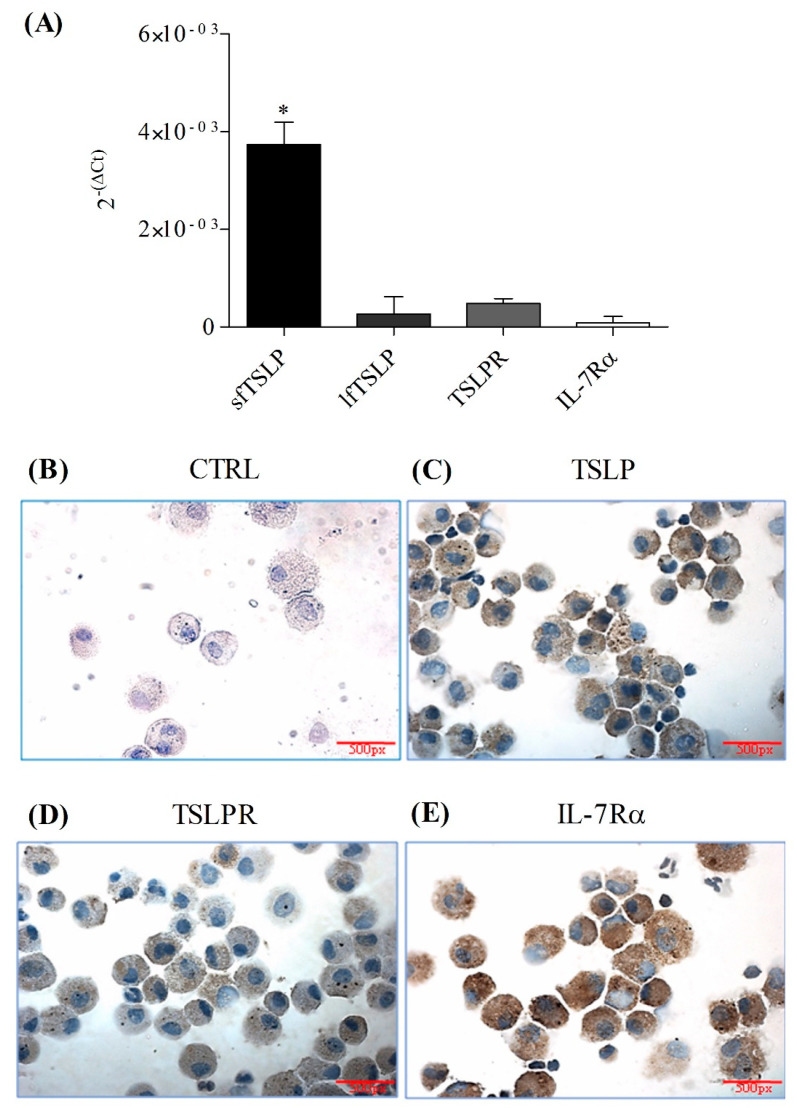
Constitutive expression of TSLP system in human lung macrophages (HLMs). The constitutive expression of sfTSLP, lfTSLP, TSLPR and IL-7Rα mRNAs was evaluated by quantitative RT-PCR in highly purified HLMs (4.5 × 10^6^ cells/well) (**A**). Data are mean ± SD of 6 independent experiments obtained from different patients. Cytocentrifuge preparations of HLMs were immunohistochemically stained for TSLP (**C**), TSLPR (**D**), and IL-7Rα (**E**) with specific primary antibodies or in absence (**B**) of primary antibody (CTRL) as described in Materials and Methods. Microscope magnification 60×. Results are representative of 6 independent experiments obtained from different patients. * *p* < 0.01 when compared to lfTSLP, TSLPR, IL-7Rα. px: pixels.

In a series of six different experiments, we investigated whether highly purified primary human lung macrophages (HLMs) constitutively express the TSLP receptor (TSLPR and IL-7Rα), the short-form TSLP (sfTSLP) and the long-form TSLP (lfTSLP) by different techniques. HLMs constitutively expressed sfTSLP mRNA (Figure 1A), whereas lfTSLP mRNA was barely detectable. Immunoreactive TSLP protein was detected in HLMs by immunohistochemistry (Figure 1C) and in lysed cells by ELISA (2.36 ± 1.29 pg/mg of protein). We also examined the constitutive expression of TSLPR and IL-7Rα by two different techniques. HLMs expressed low levels of TSLPR and IL-7Rα mRNAs (Figure 1A), whereas TSLPR (Figure 1D) and IL-7Rα (Figure 1E) were detected by immunohistochemistry in HLMs. In particular, IL-7Rα showed higher positive staining compared to TSLPR. Omission of the primary antibody resulted in negative staining (Figure 1B).

### 3.2. Effects of IL-4, IL-13, and LPS on TSLP System in HLMs

**Figure 2:**
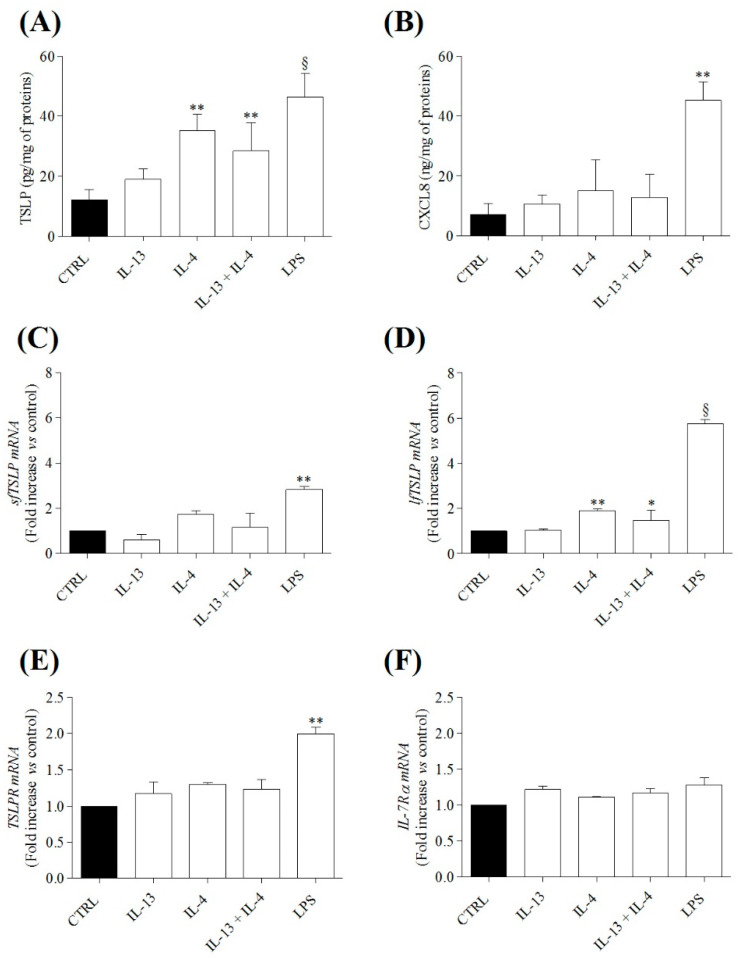
Effects of IL-13 and IL-4, alone or in combination, and of LPS on TSLP system in HLMs. Highly purified HLMs (1.5 × 10^5^ cells/well) were incubated (16 h, 37 °C) in the absence (CTRL) or in the presence of IL-13 (10 ng/mL), IL-4 (10 ng/mL) or their combination, or LPS (100 ng/mL (**A**,**B**). At the end of incubation, TSLP (**A**) and CXCL8 (**B**) proteins in supernatants were evaluated by ELISA. In parallel experiments, HLM (4.5 × 10^6^ cells/well) were incubated (6 h, 37 °C) in the absence (CTRL) or in presence of IL-13 (10 ng/mL), IL-4 (10 ng/mL) or their combination, or LPS (100 ng/mL). At the end of incubation, sfTSLP (**C**), lfTSLP (**D**), TSLPR (**E**), and IL-7Rα (**F**) mRNAs were determined by quantitative RT-PCR. Data are mean ± SD of 6 independent experiments obtained from different patients. * *p* < 0.01, ** *p* < 0.001 and ^§^ *p* < 0.0001 vs. CTRL.

We have previously shown that LPS can activate HLMs to release several proinflammatory and immunomodulatory mediators [59,75]. T_H_2-like cytokines, IL-4 and IL-13, can synergize with LPS in several systems [16]. In a series of six different experiments, we evaluated the effects of incubation (16 h, 37 °C) of IL-13 (10 ng/mL) and IL-4 (10 ng/mL), alone or in combination, and of LPS (100 ng/mL) on the release of total TSLP and of CXCL8 from HLMs. LPS was a potent stimulus for the release of both TSLP (Figure 2A) and CXCL8 from HLMs (Figure 2B). IL-4, but not IL-13, induced the release of TSLP compared to control. Both IL-4 and IL-13 did not increase the release of CXCL8 from HLMs (Figure 2B). The combination of IL-4 plus IL-13 did not increase the release of TSLP induced by IL-4 (Figure 2A). IL-4 plus IL-13 had no effect on CXCL8 production from HLMs (Figure 2B). In selected experiments, preincubation (30 min, 37 °C) of HLMs with actinomycin D (1 μg/mL), a transcription inhibitor [78], completely blocked LPS- and IL-4-induced TSLP release from HLMs (data not shown), suggesting that these stimuli caused the de novo synthesis of TSLP.

We also evaluated the effects of IL-4, IL-13, alone or in combination, and of LPS on TSLP isoforms, TSLPR, and IL-7Rα mRNAs in HLMs. LPS markedly increased the proinflammatory lfTSLP mRNA (Figure 2D), and to a lesser extent, sfTSLP mRNA (Figure 2C). IL-13, IL-4, and their combination had no effect of sfTSLP mRNA expression (Figure 2C). By contrast, IL-4, but not IL-13, upregulated lfTSLP mRNA (Figure 2D). The combination of IL-4 plus IL-13 also increased lfTSLP mRNA (Figure 2D). In parallel experiments, LPS upregulated only TSLPR mRNA (Figure 2E) but not IL-7Rα while IL-4 and IL-13, alone or in combination, had no effects on TSLPR and IL-7Rα (Figure 2E,F).

### 3.3. Effects of IL-4 and IL-13, Alone or in Combination, on Cytokine Release from LPS-Activated HLMs

**Figure 3:**
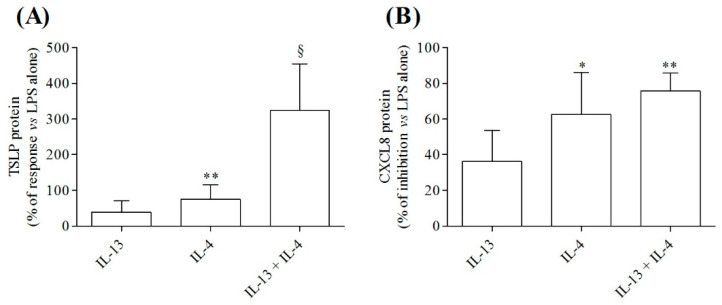
Effects of IL-13 and IL-4, alone or in combination, on TSLP and CXCL8 release from LPS-activated HLMs. Highly purified HLMs (1.5 × 10^5^ cells/well) were preincubated (10 min, 37 °C) with IL-13 (10 ng/mL) or IL-4 (10 ng/mL), alone or in combination, before the stimulation with LPS (100 ng/mL). TSLP (**A**) and CXCL8 (**B**) proteins in supernatants were evaluated by ELISA. Data are mean ± SD of 6 independent experiments obtained from different patients. * *p* < 0.01, ** *p* < 0.001 and ^§^ *p* < 0.0001 vs. LPS alone.

We next examined whether IL-4 or IL-13, alone or in combination, modified cytokine production (i.e., TSLP and CXCL8) from LPS-activated HLMs. Figure 3A shows that preincubation (10 min, 37 °C) of HLMs with IL-4 (10 ng/mL), but not IL-13 (10 ng/mL), before the stimulation with LPS (100 ng/mL) significantly potentiated TSLP release from HLMs. Although, IL-13 alone had no effect, the combination of IL-4 plus IL-13 further enhanced the production of TSLP from LPS-activated HLMs (Figure 3A). Surprisingly, IL-4 and the combination IL-4 plus IL-13 equally inhibited LPS-induced CXCL8 release from HLMs (Figure 3B). IL-13 alone had no significant effect on the release of CXCL8 from LPS-activated HLMs (Figure 3B).

### 3.4. TSLP System in Monocytes and Monocyte Macrophage-Derived (MDMs)

**Figure 4:**
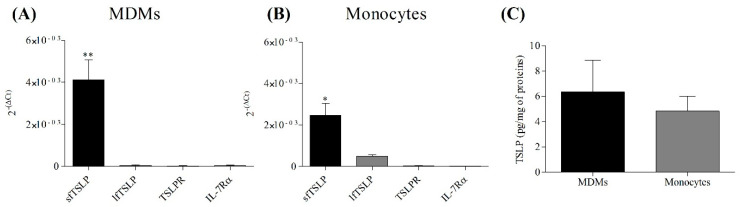
Constitutive expression of sfTSLP, lfTSLP, TSLPR, and IL-7Rα mRNAs and intracellular concentration of TSLP protein in MDMs and monocytes. MDMs (4.5 × 10^6^ cells/well) (**A**) and freshly purified monocytes (4.5 × 10^6^ cells/well) (**B**) were lysed with RNA lysis buffer to evaluate the expression of sfTSLP, lfTSLP, TSLPR and IL-7Rα mRNAs by quantitative RT-PCR (**A**,**B**). Total TSLP intracellular concentrations in MDMs and monocytes were evaluated by ELISA (**C**). Data are mean ± SD of 6 independent experiments obtained from different healthy donors. * *p* < 0.05 when compared to lfTSLP, TSLPR, IL-7Rα. ** *p* < 0.01 when compared to lfTSLP, TSLPR, IL-7Rα.

We also assessed the expression of TSLP system in another model of human macrophages such as monocyte-derived macrophages (MDMs) and on their precursors, the peripheral blood monocytes [59]. MDMs (Figure 4A) and freshly isolated monocytes (Figure 4B) constitutively expressed sfTSLP mRNA. lfTSLP, TSLPR, and IL-7Rα mRNAs were essentially undetectable in both MDMs and monocytes. Figure 4C shows that peripheral blood monocytes and MDMs contained immunoreactive total TSLP protein evaluated by ELISA.

**Figure 5:**
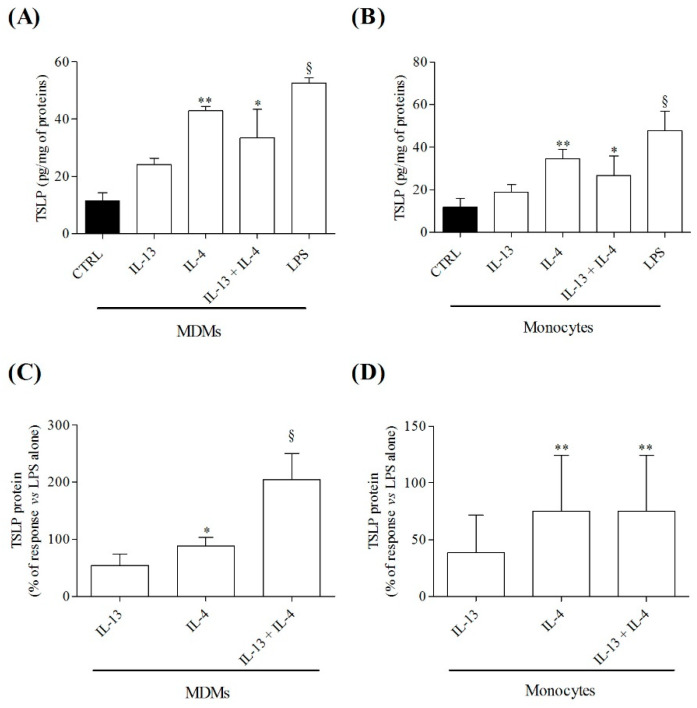
Effects of IL-13, IL-4, alone or in combination, and of LPS on TSLP production by MDMs and monocytes. MDMs (1.5 × 10^5^ cells/well) (**A**) and monocytes (1.5 × 10^5^ cells/well) (**B**) were incubated (16 h, 37 °C) in the presence of IL-13 (10 ng/mL), IL-4 (10 ng/mL), alone or in combination, or LPS (100 ng/mL). In parallel experiments, MDMs (1.5 × 10^5^ cells/well) (**C**) and monocytes (1.5 × 10^5^ cells/well) (**D**) were preincubated (10 min, 37 °C) with IL-13 (10 ng/mL) or IL-4 (10 ng/mL), alone or in combination, before the stimulation with LPS (100 ng/mL). Incubation continued for 16 h at 37 °C. In both groups of experiments, at the end of incubations TSLP concentrations in supernatants were evaluated by ELISA. Data are mean ± SD of 6 independent experiments obtained from different healthy donors. * *p* < 0.01, ** *p* < 0.001 and ^§^ *p* < 0.0001 vs. CTRL (**A**,**B**) and vs. LPS (**C**,**D**).

We next evaluated the effects of IL-4 (10 ng/mL) and IL-13 (10 ng/mL), alone or in combination, and of LPS (100 ng/mL), on TSLP system in MDMs and monocytes. Similarly to HLMs, both LPS and IL-4 induced the release of TSLP from both MDMs (Figure 5A) and monocytes (Figure 5B), whereas IL-13 had no effect. Preincubation (10 min, 37 °C) of MDMs and monocytes with IL-4, but not IL-13, enhanced TSLP production from LPS-activated MDMs (Figure 5C) and monocytes (Figure 5D). The combination of two cytokines, IL-4 plus IL-13, did not enhance the activating property of IL-4 on MDMs (Figure 5C) and on peripheral blood monocytes (Figure 5D).

### 3.5. Effects of TSLP on the Release of Angiogenic and Lymphangiogenic Factors from HLMs

**Figure 6:**
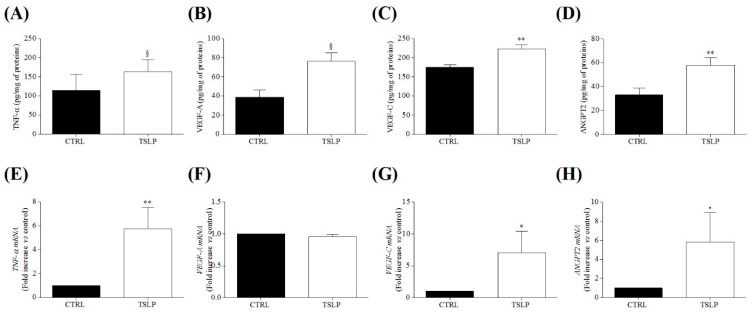
Effects of TSLP on the release and expression of inflammatory, angiogenic and lymphangiogenic mediators from HLMs. HLMs (1.5 × 10^5^ cells/well) were incubated (16 h, 37 °C) in the absence (CTRL) or in the presence of TSLP (5 ng/mL). At the end of the incubation, TNF-α (**A**), VEGF-A (**B**), VEGF-C (**C**) and ANGPT2 (**D**), and concentrations in the supernatants were evaluated by ELISA. In parallel experiments, HLMs (4.5 × 10^5^ cells/well) were incubated (6 h, 37 °C) in the absence (CTRL) or in the presence of TSLP (5 ng/mL). TNF-α (**E**), VEGF-A (**F**), VEGF-C (**G**), and ANGPT2 (**H**) mRNAs were determined by quantitative RT-PRC. Data are mean ± SD of 4 independent experiments obtained from different patients. ^§^ *p* < 0.0001 vs. control (CTRL). * *p* < 0.05 vs. control; ** *p* < 0.01 vs. control (CTRL).

Our results show that HLMs constitutively express the TSLP receptors and contain TSLP, which can be immunologically released. These results prompted us to investigate whether HLMs could be a target of TSLP. Therefore, in four independent experiments, we assessed the effects of TSLP on the release of inflammatory, angiogenic, and lymphangiogenic mediators from HLMs. Figure 6 shows that incubation (24 h, 37 °C) of HLMs with TSLP (5 ng/mL) induced the release of proinflammatory TNF-α (Figure 6A), angiogenic (VEGF-A and ANGPT2) (Figure 6B,C), and lymphangiogenic (VEGF-C) mediators (Figure 6D). The release of TNF-α, VEGF-C and ANGPT2 induced by TSLP was mediated by the activation of gene transcription (Figure 6E,G,H). Interestingly, TSLP did not induce the expression for VEGF-A mRNA (Figure 6H), suggesting that VEGF-A is released from intracellular stores.

### 3.6. Expression of TSLP System in Peritumoral and Intratumoral Human Lung Cancer

**Figure 7:**
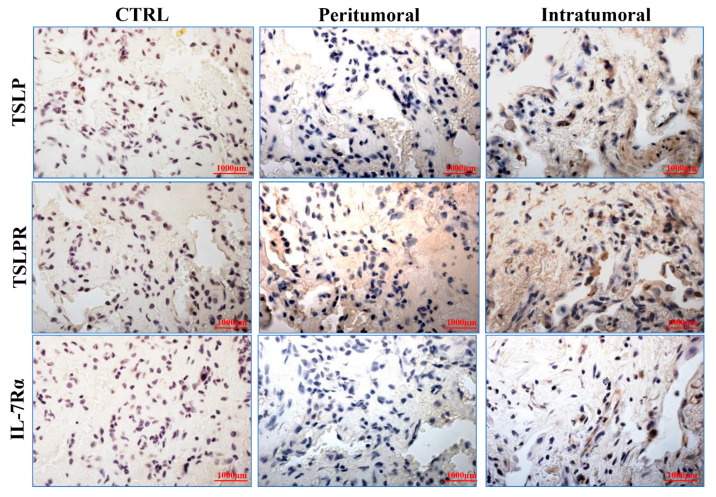
Expression of TSLP, TSLPR, and IL-7Rα in peritumoral and intratumoral human lung cancer by immunohistochemistry. Immunohistochemical staining for TSLP, TSLPR, and IL-7Rα in peritumoral and intratumoral human lung cancer. In the control (CTRL), the primary antibody was omitted. Microscope magnification 20×. This experiment is representative of 5 experiments obtained from different patients.

TSLP is a pleiotropic cytokine that has been implicated in a variety of immune disorders, including different solid and hematologic tumors [2]. The role of TSLP in cancer is rather controversial [2], although in the majority of tumors it plays a protumorigenic role [33,41,44,45]. We evaluated the expression of TSLP, TSLPR, and IL-7Rα by immunohistochemistry in peritumoral and intratumoral areas of human lung cancer (Figure 7). The results of a typical experiment showed that the expression of TSLP, TSLPR and IL-7Rα was higher in the intratumoral area compared to peritumoral area of lung cancer. Similar results were obtained in five independent experiments.

**Figure 8:**
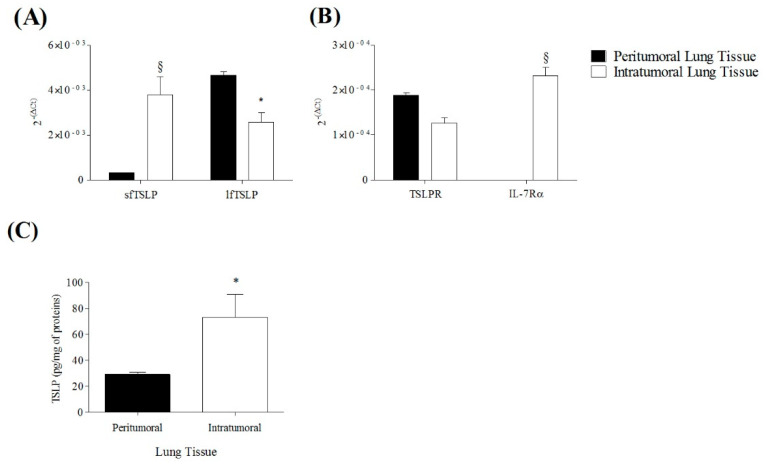
Expression of sfTSLP, lfTSLP, TSLPR, and IL-7Rα mRNAs in peritumoral and intratumoral human lung tissues. Peritumoral and intratumoral human lung tissues (2 mg) disrupted by homogenization were lysed and 500 µL of 0.1% Triton X-100. sfTSLP and lfTSLP mRNAs were determined by quantitative RT-PCR (**A**,**B**). Total TSLP was evaluated in lysed peritumoral and intratumoral lung cancer tissue by ELISA (**C**). Data are mean ± SD of 5 independent experiments obtained from different patients. * *p* < 0.01 and ^§^ *p* < 0.0001 vs. peritumoral lung tissue.

To confirm and extend the previous observation, we evaluated the expression of sfTSLP, lfTSLP, TSLPR, and IL-7Rα mRNAs in intratumoral and peritumoral areas of human lung cancer. In a series of five different experiments, the anti-inflammatory sfTSLP mRNA was significantly more expressed in the intratumoral area compared to the peritumoral region. The proinflammatory lfTSLP mRNA isoform was highly present in both peritumoral and intratumoral tissues, but significantly more expressed in peritumoral section (Figure 8A). TSLPR mRNA was equally distributed in peri- and intratumoral areas (Figure 8A,B), whereas IL-7Rα mRNA was detected only in intratumoral lung tissue (Figure 8B). The concentration of immunoreative total TSLP protein was higher in intratumoral lung cancer compared to peritumoral tissue (Figure 8C).

## 4. Discussion

In the present study, we have demonstrated that the TSLP system is constitutively expressed in macrophages purified from lung tissue of patients with lung cancer in monocyte-derived macrophages (MDMs) and in peripheral blood monocytes obtained from normal donors. HLMs, MDMs, and monocytes constitutively expressed the anti-inflammatory sfTSLP mRNA and contained immunoreative total TSLP protein. Incubation of HLMs with TSLP induced the release of proinflammatory (TNF-α), angiogenic (VEGF-A and ANGPT2), and lymphangiogenic (VEGF-C) factors. sfTSLP and lfTSLP were differentially expressed in peritumoral and intratumoral human lung cancer tissues.

LPS was found a potent stimulus for the release of total TSLP protein from HLMs. TSLP activated the cells by binding to a heterodimeric complex composed of TSLPR and IL-7Rα. LPS induced an increase of gene expression of TSLPR from HLMs, but had no effect on IL-7Rα expression. Interestingly, canonical T_H_2-like cytokines, IL-4 and IL-13, differently modulated the release of TSLP from HLMs. While IL-4 was a potent stimulus for the release of TSLP from HLMs, IL-13 alone was essentially ineffective. Moreover, the combination of IL-4 plus IL-13 did not increase the activating property of IL-4. These observations are rather interesting for several reasons. First, they suggest that IL-4 plays a modulatory role on the release of TSLP from primary lung macrophages. These cells are primarily involved in the pathogenesis of several lung inflammatory disorders [79], including COPD [80], and lung cancer [81,82,83]. Therefore, the interaction between IL-4 and TSLP could contribute to the development of these lung disorders.

IL-4, but not IL-13, synergistically potentiated the release of total TSLP protein induced by LPS from HLMs. The latter observation extends previous results demonstrating that IL-4 synergistically enhanced the production of TSLP induced by dsDNA from airway epithelial cells [4]. Similarly, IL-4 synergized with LPS in the expression and production of TSLP from dendritic cells (DCs) [16].

It is well established that low-grade inflammation plays a role in the switch between dormancy and proliferation of metastatic cells [84,85]. LPS nasal instillation in mice bearing dormant cancer cells caused awakening of tumor cells and cancer progression [86]. On the other side, tumors displaying a T_H_2 signature have a worse prognosis than that of tumors with T_H_1 predominant response [33,87]. De Monte and collaborators have demonstrated in human pancreatic cancer that TSLP drives the differentiation of T_H_2 cells and is associated with a worse prognosis [33]. Our results showing a synergistic interaction between IL-4 and LPS on the release of TSLP from human lung macrophages might have translational relevance in the context of lung cancer.

It is presently unclear why IL-13, which shares many [88,89] but not all immunological and biological effects with IL-4 [90,91], did not induce the release of TSLP from HLMs and did not potentiate the activating property of LPS. IL-4 and IL-13 are encoded by adjacent genes that share many *cis*-acting and trans-activating regulatory elements, and they signal through a partially shared receptor and adaptor system [92,93]. In particular, IL-4 activates the type I (IL-4Rα and γc) and type II (IL-4Rα and IL-13Rα1) receptors, whereas IL-13 binds only to IL-13Rα1 chain of type II receptor and to the single chain receptor IL-13Rα2 [88,90,91,94]. In addition, epigenetic and functional studies have suggested unique and non-redundant roles for these cytokines in vivo and in vitro studies [95,96,97]. Therefore, it is not surprising that the two cytokines exert distinct effects in certain immune cells [88]. Differences between IL-4 and IL-13 have been reported on the mouse macrophages responsiveness to TSLP [98]. Whatever the interpretation of these results, given the relevance of TSLP [99,100,101,102,103,104] and T_H_2 cytokines in the pathogenesis of asthma [105,106], it is likely that these observations have translational relevance contributing to clinical manifestations of chronic inflammatory lung disorders.

Another unexpected finding of our study was the specificity of the synergistic interaction between IL-4 and LPS on the production of TSLP from HLMs. To our surprise, we found that IL-13 inhibited the release of CXCL8 from LPS-activated HLMs. Also in these experiments the modulating effect of IL-4 diverged from that of IL-13. Moreover, the combination of IL-4 plus IL-13 did not enhance the inhibitory effect of IL-4 on CXCL8 release from lung macrophages. The opposing effects of IL-13 and of IL-4 on the release of TSLP and CXCL8 from LPS-activated HLMs are intriguing but difficult to explain and deserve further studies.

Peripheral blood monocytes and tissue macrophages represent two distinct branches of the mononuclear system [52]. MDMs are derived from monocytes differentiated to macrophages in the presence of G-CSF [59]. We have previously reported some biological and immunological differences between primary HLMs and MDMs [59]. In this study, HLMs, MDMs and peripheral blood monocytes contained immunoreactive TSLP and constitutively expressed sfTSLP mRNA. In addition, in HLMs, MDMs and monocytes IL-4, but not IL-13, and LPS induced the expression of sfTSLP mRNA and the release of total TSLP. Peripheral blood monocytes and MDMs show some similarities with HLMs purified from lung cancer with respect to the TSLP system. However, a better comparison of the TSLP system should be performed among peripheral blood monocytes, MDMs and HLMs obtained from the same lung cancer patients. Collectively, these results emphasize the relevance of TSLP system in the human mononuclear phagocyte system.

The production of TSLP by HLMs suggested to us possible autocrine effects on these cells which are the most representative immune cells in human lung parenchyma [71,107]. We found that HLMs incubated with TSLP released significant amount of proinflammatory TNF-α and of several angiogenic (i.e., VEGF-A and ANGPT2) [108,109] and lymphangiogenic molecules (i.e., VEGF-C) [110,111]. These findings were supported by the observation that TSLP markedly increased the expression of TNF-α, VEGF-C, ANGPT2 mRNAs. VEGF-A mRNA was not induced by TSLP, suggesting that this angiogenic factor is contained in HLMs [75]. It has been previously shown that TSLP derived from human and mouse tumors induced VEGF-A release from alveolar macrophages and enhanced metastasis formation [30]. The release of angiogenic and lymphangiogenic mediators from TSLP-activated macrophages might explain, at least in part, the protumorigenic role in TSLP in several human cancers [31,33,35,40,41,44,45].

All the previously mentioned mediators play pivotal roles in tumor initiation and progression, tumor angiogenesis, and the formation of metastasis [109,112]. These results prompted us to investigate the expression of TSLP system in human lung cancer. Our results provide evidence, to our knowledge for the first time, that TSLP isoforms, TSLPR, and IL-7Rα were expressed in both intratumoral and peritumoral lung cancer tissues. The concentration of total TSLP protein was higher in the intratumoral area compared to peritumoral tissue. Interestingly, the proinflammatory lfTSLP mRNA isoform was highly expressed in peritumoral microenvironment of human lung cancer. The latter findings extend previous observations demonstrating that cancer cells can release TSLP [31,44,45] and that tumor cells can express TSLPR and IL-7Rα [31]. Interestingly, the anti-inflammatory and homeostatic sfTSLP was more expressed in intratumoral tissue compared to peritumoral area.

The expression of total TSLP protein has been previously described in human lung carcinoma [46]. TSLP was overexpressed intratumorally compared to peritumoral lung cancer tissue and benign lesions. Interestingly, the number of Foxp3^+^ Tregs in lung cancer tissue was significantly increased compared to peritumoral lung tissue. Finally, the authors found that TSLP activated dendritic cells favoring the differentiation and migration of CD4^+^ CD25^+^ Treg cells. It is well established that Treg cells are increased in tumor microenvironment [113,114]. Collectively, these results highlight a novel mechanism by which TSLP, produced by tumor and immune cells (e.g., macrophages), might amplify an immunosuppressive microenvironment in lung cancer.

The role of TSLP in cancer is still controversial [2,115]. Although the majority of experimental [29,30,31,32] and human studies [33,34,35,36,37,38,39,40,41,42,43,44,45,46] have demonstrated a protumorigenic role for TSLP, few groups have reported a tumor-suppressing role for TLSP in experimental [47,48,49,50,51] and human cancer [48]. These apparently contrasting results could be explained by the use of different experimental models, different types and stages of cancer, and many other reasons. We would like to suggest that the two isoforms of TSLP (lf and sfTSLP), exerting opposing effects on various aspects of inflammation [1,13,14,116], could contribute, at least in part, to explain some of these contrasting results. Further studies on the roles of TSLP isoforms and their localization in peritumoral and intratumoral areas of tumors could help clarify the TSLP role in different cancers.

TSLP is an upstream cytokine primarily released by airway epithelial cells in response to a variety of environmental stimuli [1,117], initiating a range of downstream inflammatory pathways [117]. In patients with asthma, TSLP drives a T2 lung inflammatory response [18,116], but it is also involved in non-T2 processes [117]. Our results indicate that the T_H_2 cytokine IL-4 selectively induced the release of total TSLP from HLMs. In addition, IL-4 and the combination of IL-4 plus IL-13 enhanced TSLP release from LPS-activated HLMs. The interaction between a non-IgE-mediated stimulus, such as LPS, T_H_2 cytokines and TSLP in human lung macrophages greatly extend the potential proinflammatory roles of TSLP in inflammatory lung disorders. The relevance of TSLP-dependent T_H_2 inflammation in allergic disorders and in cancer has been recently emphasized [2,28].

This study has several limitations that should be pointed out. The in vitro experiments were performed using primary macrophages obtained from lung parenchyma of patients with lung adenocarcinoma. The possibility that the underlying disease may have affected some of our results cannot be dismissed. HLMs, obtained from lung tissue, are in close proximity to cancer cells. The in vivo exposure to tumor microenvironment may have affected the expression of TSLP system and the functional activity of lung macrophages. Moreover, recent studies have highlighted the extraordinary heterogeneity of human lung macrophages [54,70,79]. Our experiments were performed using highly purified macrophages obtained from mechanically dispersed lung parenchyma. We cannot exclude the possibility that different clusters of human lung macrophages selectively express TSLP receptor and/or preferentially express sfTSLP and lfTSLP. Similarly, three subsets of human monocytes (classical, intermediate, and non-classical) have been phenotypically identified [118]. We have provided preliminary evidence of the differential expression of TSLPR in functionally discrete subsets of human monocyte [76]. Finally, there is compelling evidence of the dichotomy of two isoforms of TSLP (sf and lfTSLP) in different pathophysiological conditions [13,14,119]. In this study we measured total TSLP by ELISA because specific antibodies to identify the two isoforms are not yet available. However, we identified the two TSLP isoforms in HLMs, MDMs and monocytes, and in human lung cancer tissue. Future studies should go deeper inside the biochemical and immunological mechanisms of formation of different TSLP isoforms and their multifaceted roles in cancer and in chronic inflammatory disorders.

## 5. Conclusions

In conclusion, primary human lung macrophages, MDMs, and peripheral blood monocytes express the homeostatic sfTSLP, TSLPR, and IL-7Rα and contain immunoreactive total TSLP protein. LPS and IL-4, alone or in combination, lead to an increase of lfTSLP mRNA expression and the release of TSLP from HLMs and MDMs. TSLP induces the release of several angiogenic and lymphangiogenic factors from HLMs. TSLP protein and TSLP isoforms are found in intratumoral and peritumoral human lung cancer. Collectively, our results indicate that the TSLP system, widely expressed throughout the human mononuclear system, could be involved in chronic inflammatory disorders and lung cancer.

## Data Availability

Data supporting the reported results are available upon request.

## Abbreviations

CAF	cancer-associated fibroblast
DC	dendritic cell
FCS	fetal calf serum
HBsAg	hepatitis B surface Ag
HLM	human lung macrophage
IL	interleukin
IL-7Rα	interleukin-7 receptor α
lfTSLP	long form TSLP
LPS	lipopolysaccharide
MDM	monocyte-derived macrophage
PAP	peroxidase anti-peroxidase
PBMC	peripheral blood mononuclear cell
RT-PCR	quantitative reverse transcriptase PCR
sfTSLP	short form TSLP
T_H_2	T helper 2
TSLP	thymic stromal lymphopoietin
TSLPR	thymic stromal lymphopoietin receptor
VEGF	vascular endothelial growth factor
